# Supplementary material for: Incommensurate smectic phase in close proximity to the high-Tc superconductor FeSe/SrTiO3
Source: Nat Commun. 2021 Apr 13;12:2196. doi: 10.1038/s41467-021-22516-2 (PMC8044195; doi:10.1038/s41467-021-22516-2)
Supplement: Supplementary file 1 — Supplementary Information [file 41467_2021_22516_MOESM1_ESM.pdf]

Supplementary information

Incommensurate smectic phase in close proximity to the high-T_c superconductor $FeSe/SrTiO_3$

Yonghao Yuan[1,2], Xuemin Fan[1,2], Xintong Wang[1,2], Ke He[1,2,3], Yan Zhang[4,5], Qi-Kun Xue[1,2,3]* and Wei Li[1,2]*

[1]*State Key Laboratory of Low-Dimensional Quantum Physics, Department of Physics, Tsinghua University, Beijing 100084, China*

[2]*Frontier Science Center for Quantum Information, Beijing 100084, China*

[3]*Beijing Academy of Quantum Information Sciences, Beijing 100193, China*

[4]*International Centre for Quantum Materials, School of Physics, Peking University, Beijing 100871, China*

[5]*Collaborative Innovation Centre of Quantum Matter, Beijing 100871, China*

*To whom correspondence should be addressed: weili83@tsinghua.edu.cn; qkxue@mail.tsinghua.edu.cn

Supplementary Note 1: The 2×1 reconstruction in 1 UC FeSe

Supplementary Fig. 1a shows a topographic image of an area including 1 UC and 2 UC FeSe. The 2×1 domain boundaries (see the white dashed lines in Supplementary Fig. 1a) are clearly resolved and they continuously cross the step edge. A zoomed-in image on one of the boundary regions (denoted by the red rectangle in Supplementary Fig. 1a) is presented in Supplementary Fig. 1b. The 2×1 domains show 90° rotation on the two sides of the domain boundary, which can be clearly seen in their corresponding fast Fourier transformation images (Supplementary Fig. 1c, d). The 2×1 spots are denoted by the red dashed circles.

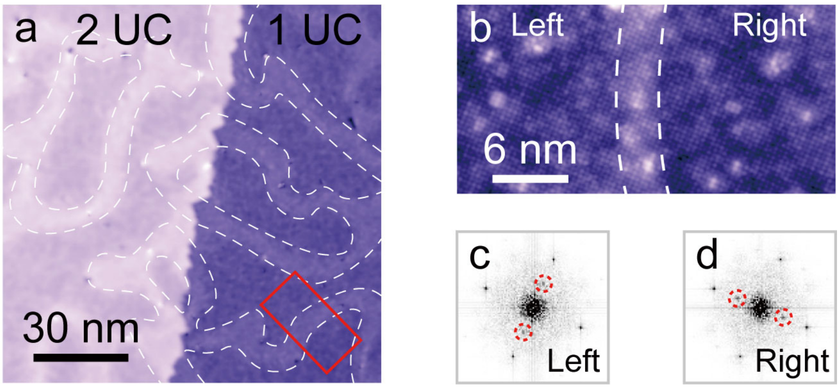

Supplementary Figure 1 The 2×1 reconstruction in 1 UC FeSe. **a** STM topographic image of an area including 1 UC and 2 UC FeSe (120 nm×120 nm; set point, V_s = 2.0 V, I_t = 20 pA). The 2×1 domain boundaries (white dashed line) continuously cross the step edge. **b** STM topographic image of a 2×1 domain boundary of 1 UC FeSe. Its location in **a** is denoted by the red rectangle (30 nm×15 nm; set point, V_s = 60 mV, I_t = 300 pA). The domain regions on the left and right sides of the domain boundary show 90° rotation of the 2×1 reconstruction. **c**, **d** Fast Fourier transformation images of the left and right domains in **b**. The red dashed circles denote the 2×1 spots.

Supplementary Note 2: Raw d*I*/d*V* data in Fig. 1d

Supplementary Fig. 2a shows the raw d*I*/d*V* data in Fig. 1d. The spectra in different colors correspond to the different energy ranges in Fig. 1d. The gray dashed lines highlight the peak and dip positions of the stripe ordering. It is hard to make a direct analysis for these raw data, because the oscillation amplitudes of the spectra are significant different from each other. In order to give a complementary analysis other than calculating the d^3I/dV^3 that is shown in the main text, each spectrum is divided by its linear background, re-scaled by multiplying a factor, shifted for clarity and finally shown in Supplementary Fig. 2b (the corresponding energies and scaling factors are labeled on the right of the spectra). It shows that the period of the stripes doesn't change with energy and the stripes in 30 meV to 60 meV have a π phase shift. These

conclusions are consistent with those obtained from the d^3I/dV^3 spectra in Fig. 2d.

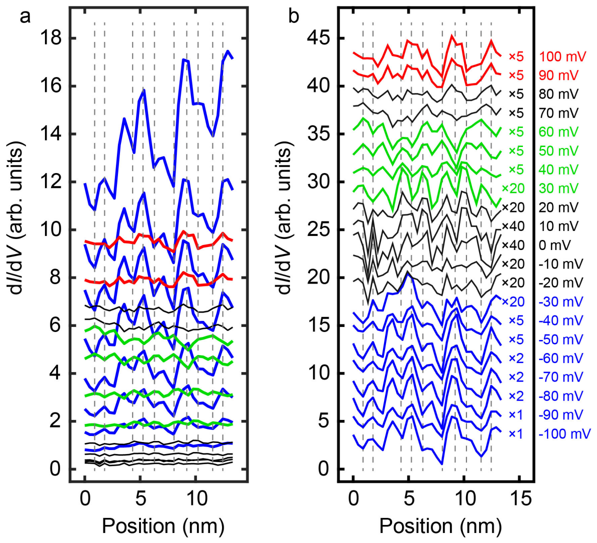

Supplementary Figure 2 d*I*/d*V* line-cut in Fig. 1d. **a** Raw d*I*/d*V* spectra. **b** The normalized d*I*/d*V* spectra. The spectra are shifted for clarity. The red, green and blue colors correspond to the different energy ranges in Fig. 1d. The gray dashed lines denote the peak and dip positions in the spectra.

Supplementary Note 3: Evidence of nematicity: Quasiparticle interference (QPI) in 3 UC FeSe

Strong nematicity in FeSe thin films leads to the lift of degeneracy between the d_{xz} and d_{yz} bands [1,2]. STM can also detect such band separation by the inequivalent QPI patterns along the *a*- and *b*-directions [3,4].

In order to obtain the symmetry breaking information and determine the *a*- and *b*-directions in 3 UC FeSe, QPI measurement is carried out in the vicinity of defects. Two Fe-vacancies are introduced as scattering centers (Supplementary Fig. 3a), in which short-range stripes are clearly revealed. The unidirectional QPI patterns above E_F are shown in Supplementary Fig. 3b-k and indicated by magenta arrows. The QPI patterns are along the diagonal direction of Se-Se lattice (corresponds to the direction of Fe-Fe lattice) and break C4 symmetry in real-space. They propagate along the direction of the short-rang stripes. The patterns move towards the scattering centers at higher energies, indicating a shorter wavelength in real-space (or a larger scattering wave vector in *q* space). Such an energy dispersive behavior corresponds to an electron-like band above E_F. The scattering wave vector q_b is extracted from each image and fitted by a parabolic curve (Supplementary Fig. 3l), indicating that the electron-like band reaches its band bottom at around 60 meV above E_F.

The QPI results also exhibit unidirectional patterns below E_F (Supplementary Fig. 4a-h),

but they are along the perpendicular direction to those taken above E_F. The QPI patterns show two ring-like features in the vicinity of defects (denoted by the yellow dashed rings in Supplementary Fig. 4b). The scattering wavelength is determined by the far end-points of the ring features (the yellow arrows in Supplementary Fig. 4a-h), which become larger with energy closer to E_F, reflecting a scattering process from a hole-like band. Figure 4i shows the extracted scattering wave vector q_a and the corresponding parabolic fitting result, which indicates that the hole-like band reaches its band top at around -15 meV.

The energy-dependent quasiparticle interference patterns of 3 UC FeSe are more clearly shown in Supplementary Movie 1 and 2.

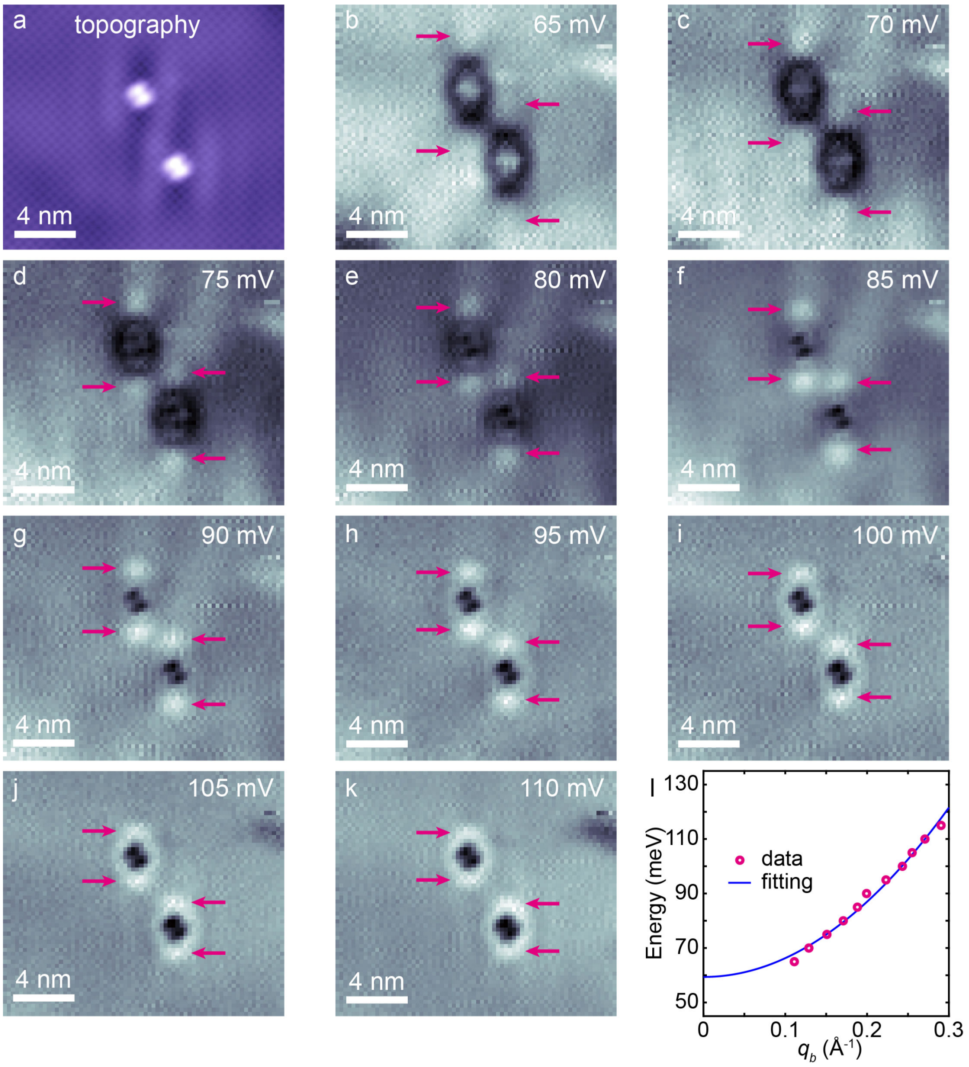

Supplementary Figure 3 Quasiparticle interference in 3 UC FeSe at positive energy. **a** STM topographic image of two Fe-vacancies in 3 UC FeSe film (13.75 nm × 12 nm; set point, V_s = 60 mV, I_t = 400 pA). **b-k** Positive energy dI/dV mappings taken at the same position (13.75 nm × 12 nm; set point, V_s = 120 mV, I_t = 1 nA). The magenta arrows denote the energy-dependent QPI patterns. **l** Energy dispersion of the scattering wave vector q_b. A parabolic fitting

result is shown in a blue curve.

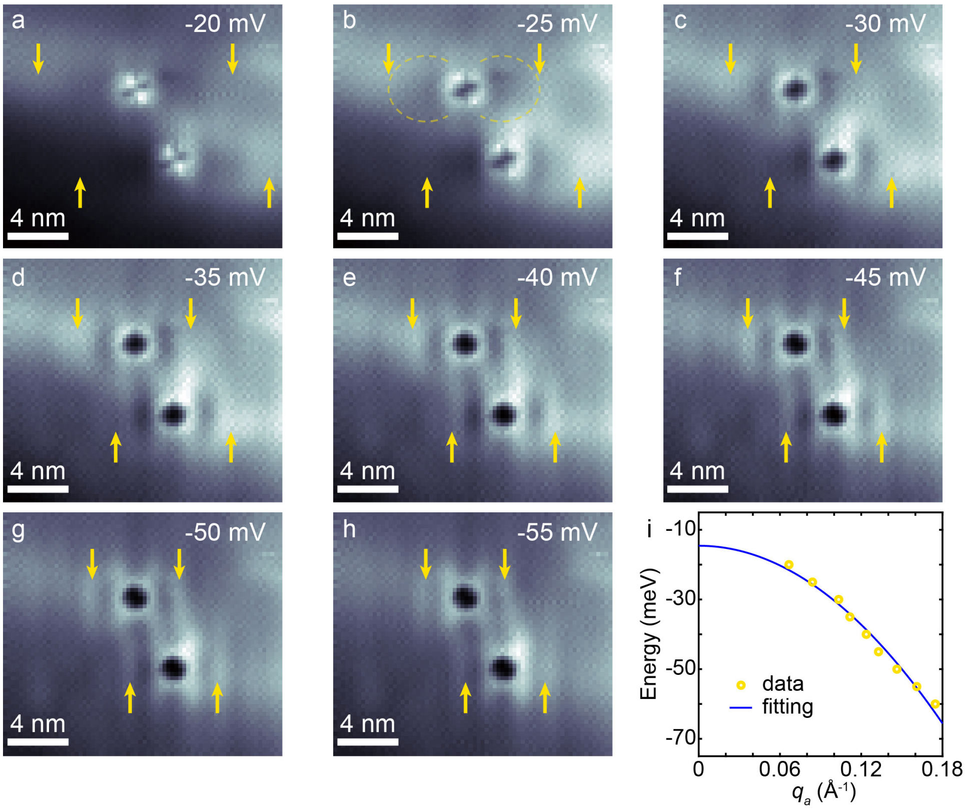

Supplementary Figure 4 Quasiparticle interference in 3 UC FeSe at negative energy. **a-h** Negative energy d*I*/d*V* mappings taken at the same position (13.75 nm × 12 nm; set point, V_s = 120 mV, I_t = 1 nA). The yellow arrows denote the energy-dependent QPI patterns. **I** Energy dispersion of the scattering wave vector q_a. A parabolic fitting result is shown in a blue curve.

Supplementary Note 4: Evidence of nematicity: Quasiparticle interference in 2 UC FeSe

The QPI measurement is also employed in 2 UC FeSe and it presents similar results to that in 3 UC FeSe. The QPI patterns at positive energies indicate a scattering process from an electron-like band and its band bottom locates above E_F (Supplementary Fig. 5). The ring-like QPI patterns at negative energies indicate the presence of a hole-like band structure and its band top locates below E_F (Supplementary Fig. 6).

The similarity of QPI results in 2 UC and 3 UC FeSe indicates the band structures in these two layers are similar, which is consistent with previous APRES data[1]. The d_{xz} and d_{yz} bands in 2 UC FeSe also have a considerable separation and inequivalence along the k_x and k_y direction.

The energy-dependent quasiparticle interference patterns of 2 UC FeSe are more clearly shown in Supplementary Movie 3 and 4.

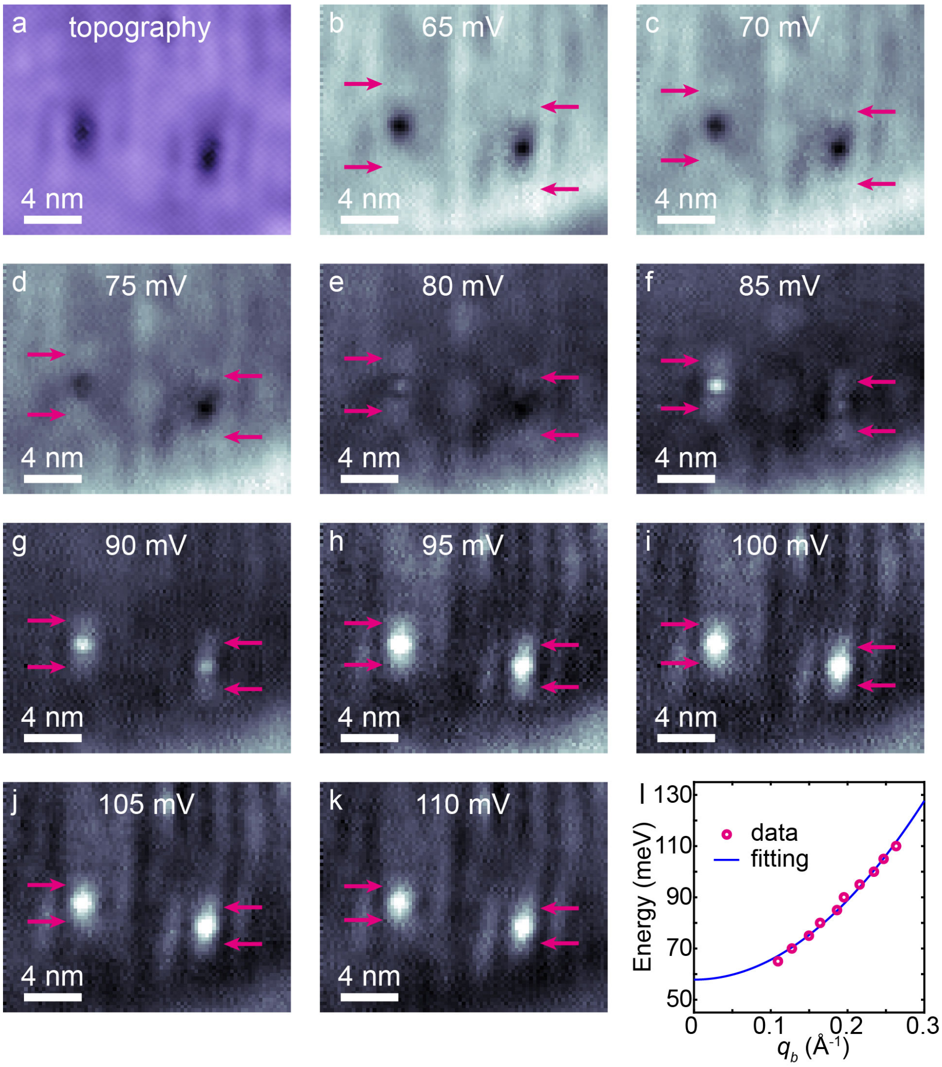

Supplementary Figure 5 Quasiparticle interference in 2 UC FeSe at positive energy. **a** STM topographic image of two defects in 2 UC FeSe film (20 nm × 16 nm; set point, V_s = 60 mV, I_t = 200 pA). **b-k** Positive energy dI/dV mappings taken at the same position (20 nm × 16 nm; set point, V_s = 120 mV, I_t = 500 pA). The magenta arrows denote the energy-dependent QPI patterns. **l** Energy dispersion of the scattering wave vector q_b. A parabolic fitting result is shown in a blue curve.

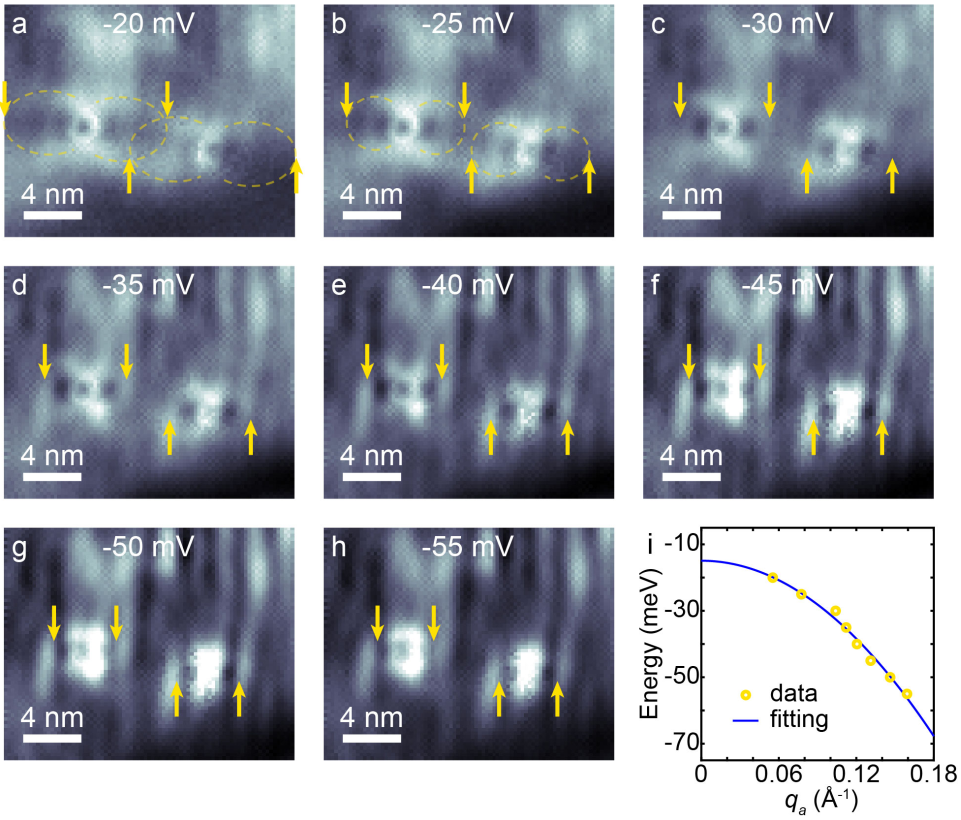

Supplementary Figure 6 Quasiparticle interference in 2 UC FeSe at negative energy. **a-h** Negative energy dI/dV mappings taken at the same position (20 nm × 16 nm; set point, V_s = 120 mV, I_t = 500 pA). The yellow arrows denote the energy-dependent QPI patterns. **I** Energy dispersion of the scattering wave vector q_a. A parabolic fitting result is shown in a blue curve.

Supplementary Note 5: Quasiparticle interference analysis with band structure

The origins of the anisotropic scattering wave vectors q_a and q_b are clarified by analyzing the band structure of FeSe. The nematicity in FeSe leads to a C2 symmetric band structure and the inequivalent Fermi surfaces around the M_x and M_y points. Supplementary Figure 7a is the schematic Fermi surface around the M_x point of FeSe thin films [1, 2]. The red, green and blue lines denote the d_{xz}, d_{yz} and d_{xy} bands, respectively. The different band structures along the cut 1 (k_x direction) and cut 2 (k_y direction) are shown in Supplementary Fig. 7b and c, which can give rise to different scattering vectors.

Along the k_x direction, the d_{xz} and d_{yz} bands cross with each other and form a Dirac-cone like feature below E_F (the X-shaped crosses formed by the green and blue lines in Supplementary Fig. 7b) [5]. In 50 UC FeSe, the "Dirac point" is located at -10 meV measured by ARPES [5]. The hole branch of this "Dirac-cone" is the possible origin of the scattering wave vector q_a (the double headed yellow arrow in Supplementary Fig. 7b). The band top value -15 meV obtained from the fitting to the scattering vector q_a is also consistent with the energy of

the "Dirac point". The little deviation of the value probably originates from the different strength of tensile strain in 3 UC and 50 UC thin films. Therefore, the scattering wave vector q_a is attributed to the inter-band scattering process between the d_{xz} and d_{yz} bands along the k_x direction.

In order to find the origin of q_b, which is perpendicular to q_a, the band structure along k_y direction is investigated (Supplementary Fig. 7c). According to the QPI results, q_b is related to an electron-like band with its band bottom above E_F. Therefore, the only candidate is the d_{yz} band (the green band in Supplementary Fig. 7c) and scattering wave vector q_b is attributed to the intra-band scattering process of d_{yz} band along the k_y direction.

We also make a more quantitative comparison between the QPI and APRES results. Supplementary Fig. 7d and e show the d_{yz} and d_{xy} bands formed Dirac-cone like band structures in 2 UC and 3 UC FeSe, which are extracted from previous ARPES data [1]. The double headed yellow arrows correspond to the scattering wave vectors q_a obtained from the QPI data taken at negative energies. The double headed arrows nicely link the hole branches of the "Dirac-cone", confirming that the scattering wave vector q_a originates from the inter-band scattering between d_{yz} and d_{xy} bands. The scattering wave vector q_b is not able to make a quantitative comparison to the d_{yz} band along the k_y direction, because APRES measurement cannot obtain the band structure above E_F.

Due to the nematic phase transition of FeSe, the Se-Se lattice is rhombic and the Fe-Fe lattice is orthorhombic. In the Fe-plane, the lattice constant along *a*-direction is larger than that along *b*-direction, but cannot be detected by STM due to the limitation of real-space resolution. The inequivalent scattering wave vectors q_a and q_b can help to determine the *a*- and *b*-directions in FeSe, and thus determine the direction of stripes [3]. Our data show that the long-range stripes in 2 UC FeSe and short-range stripes in 3 UC FeSe oscillate along the same direction to unidirectional QPI patterns at negative energies, indicating the wave vector of the stripes is along the *a*-direction. This result is consistent with the previous study on the short-range stripes in 30 UC FeSe [3].

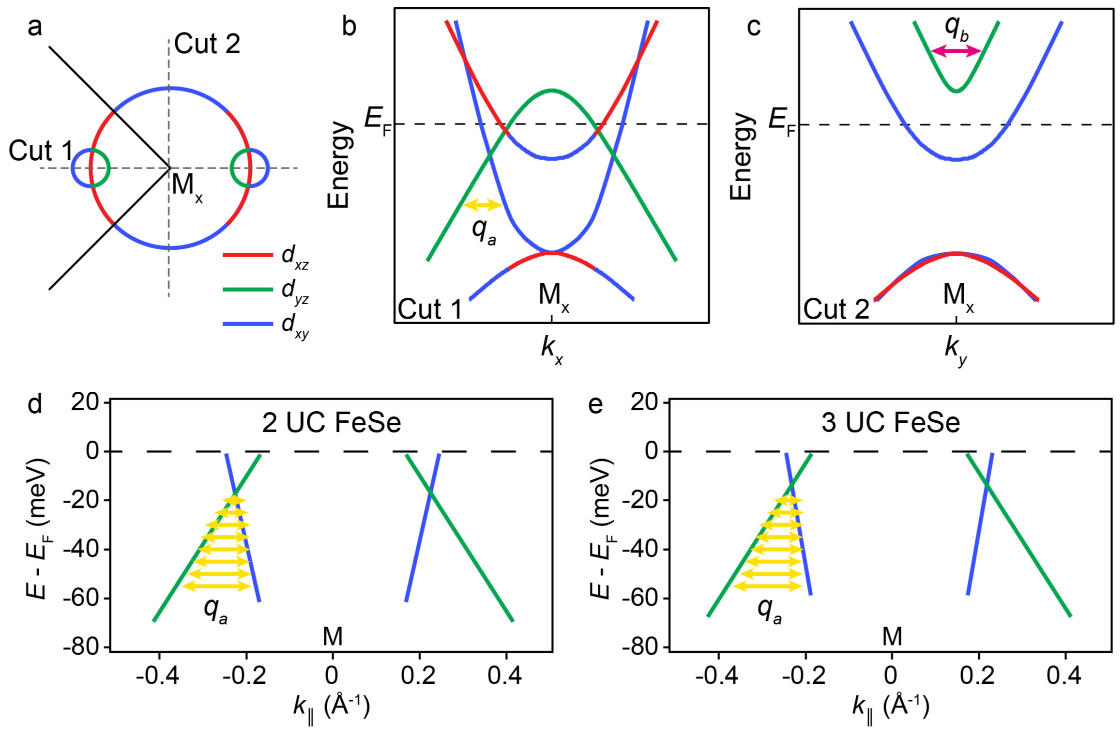

Supplementary Figure 7 Band structure and QPI. **a** Schematic Fermi surface of FeSe thin film around the M point [1, 2]. **b**, **c** Schematic band structures along the cut 1 and cut 2 in **a**, respectively [2]. The double headed yellow arrow denotes the inter-orbital scattering process between the d_{yz} and d_{xy} bands below E_F. The double headed magenta arrow denotes the intra-orbital scattering process of d_{yz} band above E_F. **d**, **e** d_{yz} and d_{xy} band structures around the M point of 2 UC and 3 UC FeSe thin films extracted from ARPES results [1]. The double headed yellow arrows are the scattering wave vectors obtained from QPI data in Supplementary Figure 4 and 6.

Supplementary Note 6: The nematic phase and short-range stripes in 3 UC FeSe

Supplementary Fig. 8a and b show topographic images of a 3 UC FeSe film obtained with different bias voltages. The nematic domain boundaries and the DOS corrugations related to the 2×1 reconstruction are simultaneously resolved (Supplementary Fig. 8a) and they are highlighted by orange dashed lines and white dashed lines, respectively (Supplementary Fig. 8b). In the vicinity of defects, short-range stripes, similar to those observed in 30 UC FeSe, can be identified in Supplementary Fig. 8b, indicating that 3 UC FeSe is in nematic phase with smectic fluctuation. The stripes are perpendicular to each other on the two sides of the nematic domain boundaries (orange dashed lines). Supplementary Fig. 8c presents a zoomed-in image of the stripes pinned by a dimer-shaped defect. These results prove that the smectic-to-nematic phase transition occurs in 3 UC FeSe and the electronic structure of 3 UC FeSe is similar to that of thicker films.

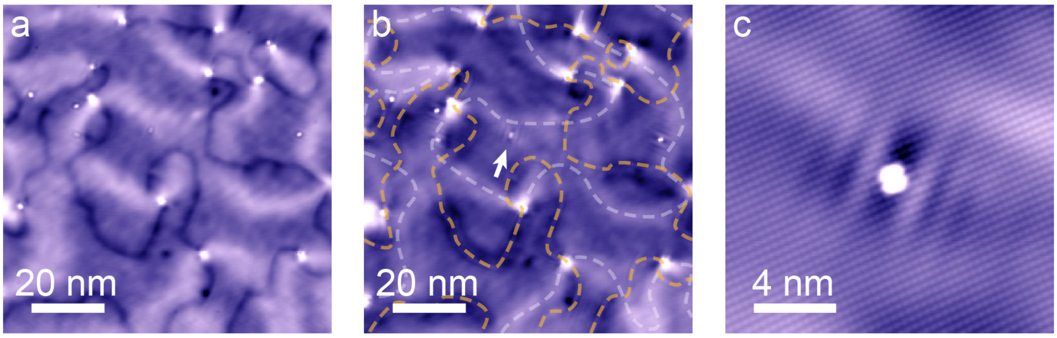

Supplementary Figure 8 The nematic phase and short-range stripes in 3 UC FeSe. **a** STM topographic image of a 3 UC FeSe film (90 nm×90 nm; set point, V_s = 60 mV, I_t = 200 pA). **b** STM topographic image on the same area of **a** (90 nm×90 nm; set point, V_s = 100 mV, I_t = 100 pA). The nematic domain boundaries and the DOS corrugations related to the 2×1 domain boundaries in 1 UC FeSe are highlighted, and they are marked by orange and white dashed lines, respectively. **c** Stripe patterns pinned by a dimer-shaped defect (16 nm×16 nm; set point, V_s = 60 mV, I_t = 400 pA). Its location in **b** is denoted by white arrow. The short-range stripes indicate the presence of smectic fluctuation.

Supplementary Note 7: 2 UC FeSe at 77 K

As shown in the STM topographic image of a 2 UC FeSe taken at 77 K (Supplementary Fig. 9), the stripe patterns disappear, but the domain walls still exist (denoted by orange dashed lines). This further supports the picture raised in the main text: the smectic phase locates beneath the nematic phase. At elevated temperature, the frozen long-range stripes are melted and the nematic electronic structure persists. The temperature evolution of the stripes is consistent with theoretical proposal and the observations in cuprates [6-8].

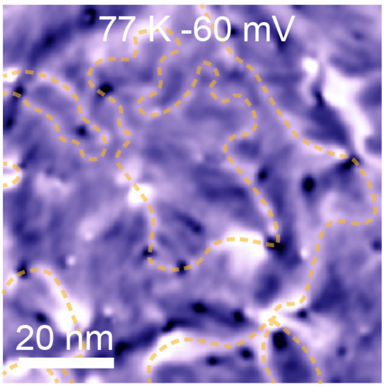

Supplementary Figure 9 Topographic image of a 2 UC FeSe/STO at 77 K (80 nm×80 nm; set point, V_s = -60 mV, I_t = 50 pA). The stripes are absent but the domain walls still exist (denoted by orange dashed lines).

Supplementary Note 8: The adsorption of single Rb atom on FeSe thin films

Supplementary Figure 10 shows an atomically resolved topographic image of single Rb atom adsorbed on 2 UC FeSe. The red dots in Supplementary Fig. 10a mark the Se sites of the Se-terminated surface. The Rb atom occupies the hollow site of the Se lattice and presents itself as different morphology at various bias voltages. When lower down the scanning bias voltage from 100 mV to 30 mV, the round Rb atom gradually separates and highlights the four nearest Se atoms, as shown in Supplementary Fig. 10a-c. At negative bias voltages, as shown in Supplementary Fig. 10d and e, the Rb atom manifests as a ring structure with weak C2 symmetry. Supplementary Figure 10f presents dI/dV spectra taken on Rb atom and bare 2 UC FeSe. Compared to that taken on bare FeSe, the dI/dV spectrum taken on Rb atom shows deviation at negative bias voltages and a wider flat terrace of density of states near E_F. These features are similar to that in 1 UC FeSe, indicating the electronic structure of 2 UC FeSe is influenced by the charge transfer from Rb atom.

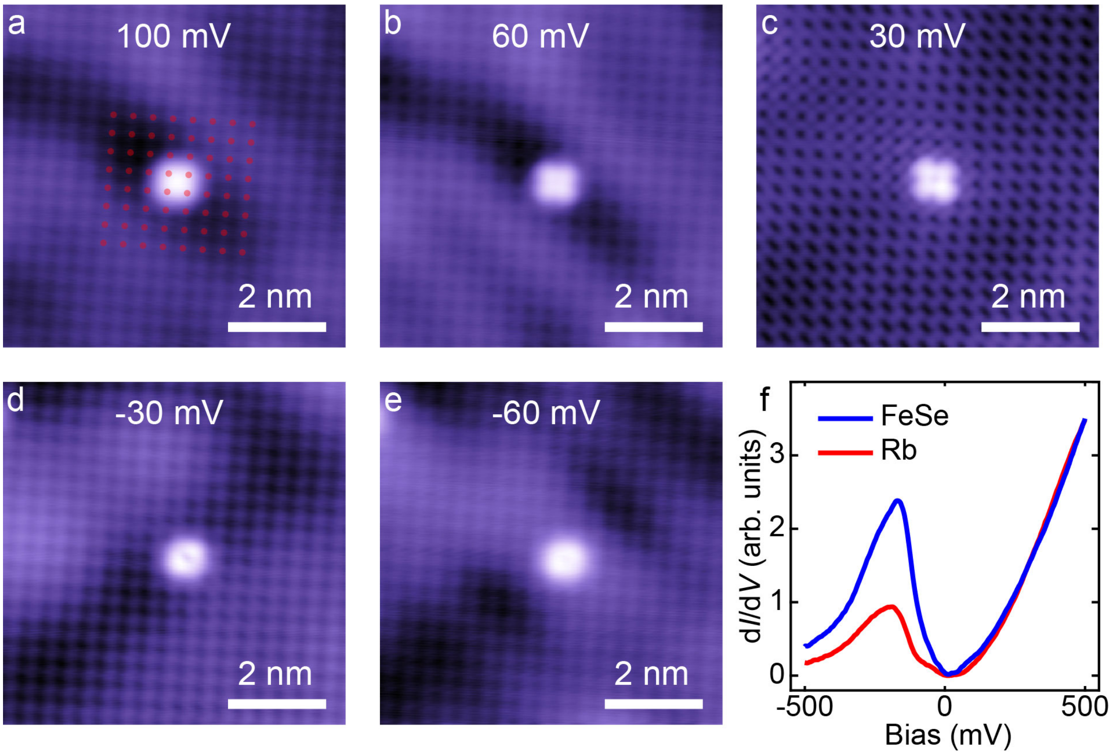

Supplementary Figure 10 Single Rb atom adsorbed on FeSe thin film. **a-e** Topographic images of a single Rb atom on 2 UC FeSe taken at different bias voltages (7 nm × 7 nm; set point, V_s = 100 mV, 60 mV, 30 mV, -30 mV, -60 mV, respectively). The red dots in **a** denote the Se sites. The Rb atom is adsorbed on the hollow site of Se lattice. **f** dI/dV spectra taken on Rb atom and bare FeSe thin film (set point, V_s = 500 mV, I_t = 200 pA).

Supplementary Note 9: Estimation of stripe area ratio

Figure 11 demonstrates the estimation of stripe area ratio at each doping level in Fig. 3. The areas where stripe ordering is absent are marked by yellow shaded regions. Stripe area ratio is estimated as A_s/A_t, where A_s is the stripe area and A_t is the total area in each image. Smectic domain walls are calculated into stripe-free regions. Therefore, the stripe area ratio of Rb-free 2 UC FeSe is not 100%.

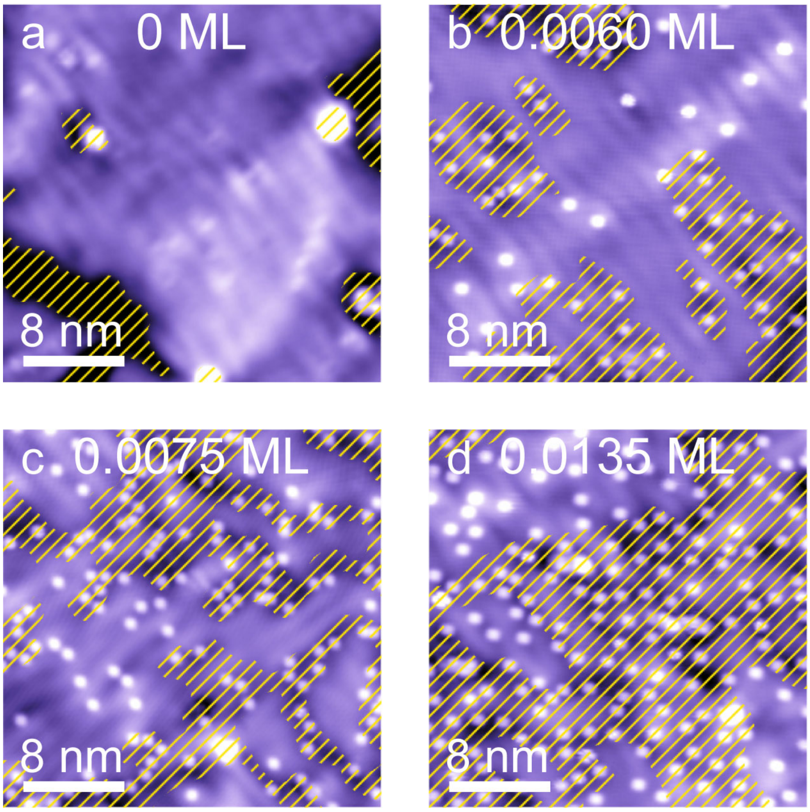

Supplementary Figure 11 Estimation of stripe area ratio. **a-d** Topographic images of 2 UC FeSe with surface Rb coverage varied from 0 to 0.0135 ML. The areas where stripe ordering is absent are marked by yellow shaded regions.

Supplementary Note 10: Topographic images of Rb coated superconducting 2 UC FeSe thin films

Supplementary Figure 12a-f show the topographic images of superconducting 2 UC FeSe, on which the Rb coverages are higher than those in Fig. 3. The topographic evolution is similar to that of K coated FeSe thin films [9, 10]. As shown in Supplementary Figure 12a, the Rb atoms randomly distribute on the surface at the coverage of 0.038 ML, at which FeSe is in underdoped regime. With the coverage increases from 0.063 ML to 0.095 ML, the Rb atoms gradually arrange close to each other and form short-range square superlattice. When the coverage

increases to 0.120 ML (Supplementary Figure 12d), at which FeSe is optimally doped, short-range hexagonal superlattice is formed and coexisted with square superlattice. The bright dots in Supplementary Figure 12d are Rb atoms adsorbed upon the first Rb atomic layer. As shown in Supplementary Figure 12e, further deposition leads to Rb clusters formed on the first Rb layer. The screening effect from the first Rb layer decreases the doping efficiency of the upper Rb. Meanwhile, the top clusters introduce inhomogeneity to the sample and decrease the superconducting ratio (Fig. 4e). At this coverage (0.145 ML), the first Rb layer still show the coexistence of square and hexagonal superlattice. Finally, when the coverage increases to 0.196 ML, the top surface is fully covered by Rb clusters, giving rise to strong inhomogeneity (Supplementary Figure 12f).

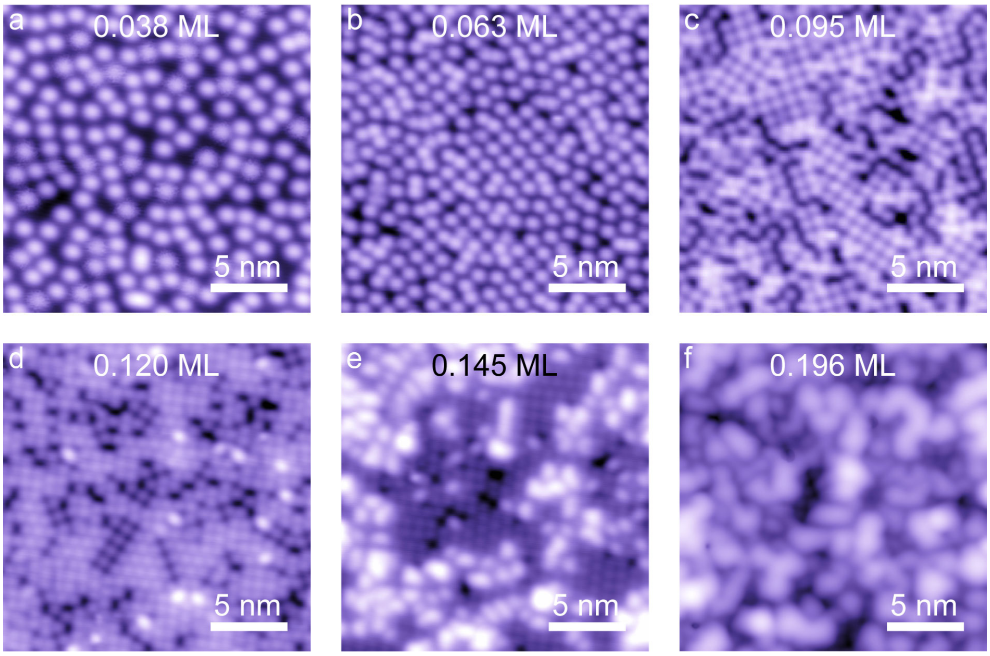

Supplementary Figure 12 a-f Topographic images of FeSe thin films with higher Rb coverages (20 nm × 20 nm).

Supplementary Note 11: Classification of good and bad superconducting gaps

The good-superconducting group contains the dI/dV spectra which show energetically symmetric (with respect to zero energy) coherence peaks and absence of in-gap states, i.e. a fully opened U-shaped gap. Supplementary Figure 13a presents three examples for good superconducting gaps. FeSe thin films usually exhibit two pairs of energetically symmetric coherence peaks (as shown in the spectra #1 and #2 in Supplementary Fig. 13a). The spectra which only have one pair of symmetric coherence peaks are also classified in good superconducting group (#3 spectra in Supplementary Fig. 13a).

The bad superconducting group includes the spectra that show asymmetric

superconducting gaps or contain in-gap states. If more than two pairs of peaks/features appear in the superconducting gap, they are recognized as in-gap states, and such spectra are sorted into bad superconducting group (see the three examples in Supplementary Fig. 13b).

In some cases, the coherence peaks and in-gap states are not symmetric in energies. For example, the spectrum #1 in Supplementary Fig. 13c shows a kink feature on the gap edge at negative energy; The spectrum #2 shows an in-gap state only at positive energy; The spectrum #3 show asymmetric coherence peaks at positive and negative energies. Such kind of spectra is also sorted into bad superconducting group.

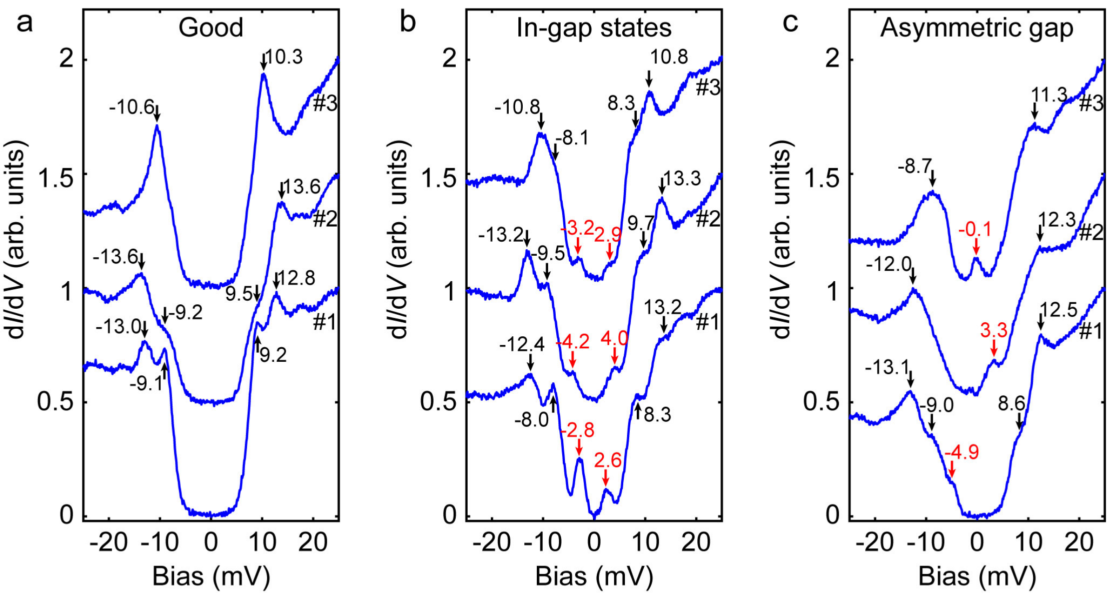

Supplementary Figure 13 Examples for good and bad superconducting gaps. **a** Three typical dI/dV spectra in good superconducting group (set point, V_s = 25 mV, I_t = 100 pA). **b, c** Examples for bad superconducting gaps which exhibit in-gap states and asymmetric features, respectively (set point, V_s = 25 mV, I_t = 100 pA). The black arrows denote the coherence peaks and the red arrows denote the in-gap states/features.

Supplementary Note 12: Comparison of the stripes to band structure

Supplementary Figure 14 shows a direct comparison between the wave vector of the stripe ordering and thickness dependent band structures of FeSe thin films measured by ARPES. Supplementary Fig. 14a is the FFT result in the inset of Fig. 1c. The wave vector length of the stripes (highlighted by red circles) is q_0 = 0.309 Å^{-1}. Supplementary Fig. 14b-f are thickness dependence of d_{yz} and d_{xy} bands around the M point extracted from ARPES data [1]. The red arrow in Supplementary Fig. 14b denotes the wave vector of the stripe ordering, which cannot link to any two bands in k-space. Therefore, the itinerate electron picture, which attribute the

origin of smectic phase to the scattering between two nesting bands, is excluded.

One may find that the d_{yz} and d_{xy} bands have two almost parallel branches near E_F, which can give rise to a possible energy-independent inter-band scattering wave vector (the wave vector q_s denoted in black double headed arrows in Supplementary Fig. 14b-f). And it might lead to the energy-independent stripe ordering. However, this scattering process is inconsistent with the stripe ordering from three aspects: (1) In 2 UC FeSe thin film (Supplementary Fig. 14b), the stripe ordering wave vector q_0 is apparently shorter than the inter-band scattering wave vector q_s = 0.406 Å^{-1}. (2) The hole-like d_{yz} band reaches its band top within 60 meV above E_F, and such inter-band scattering above this energy could not occur anymore. However, the stripe ordering can still be observed at higher energy, for example at 150 meV (Fig. 2b and f in the main text). (3) The long-range stripe orderings in 2 UC FeSe and the short-range stripe ordering in 30 UC FeSe have the same period. In contrast, the inter-band scattering wave vector q_s is thickness-dependent and shorter in thicker films.

In short, the smectic phase in 2 UC FeSe cannot be explained by an itinerate electron picture and the electronic correlation effect needs to be considered.

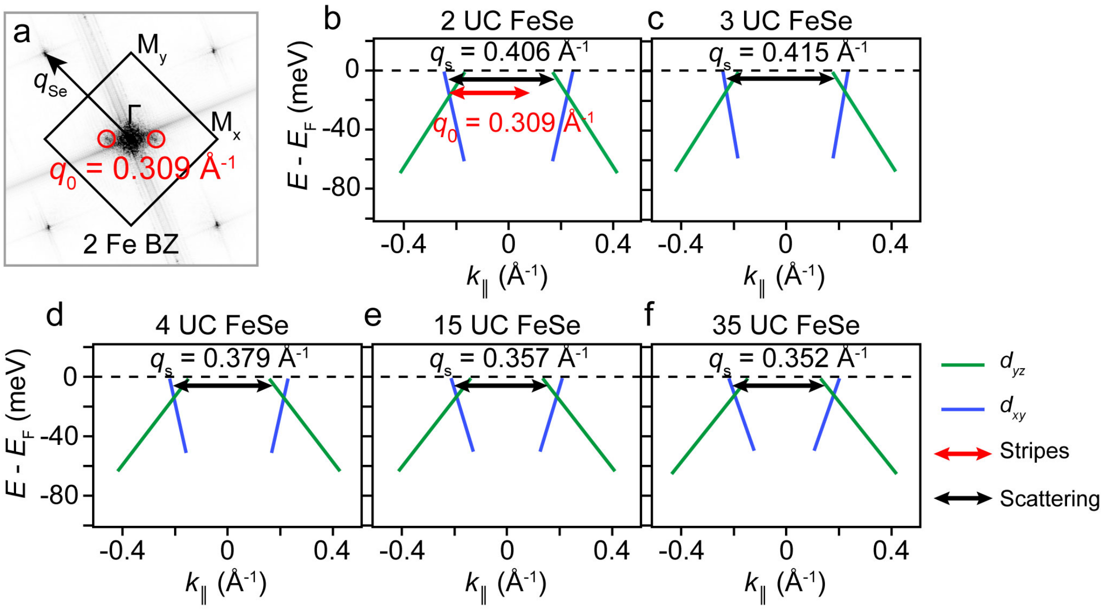

Supplementary Figure 14 Comparison of stripe ordering to band structure. **a** The FFT result in the inset of Fig 1c. The red circles highlight the scattering wave vector of the stripe ordering ($q_0 = 0.19q_{Se}$ = 0.309 Å^{-1}). The black square is the 2-Fe Brillouin zone boundary. **b-f** Thickness dependence of band structure around the M point extracted from ARPES results [1]. The blue and green lines denote the d_{xy} and d_{yz} bands in multilayer FeSe. The double headed red arrow denotes the wave vector of the stripe ordering q_0. The double headed black arrows denote the possible inter-band scattering wave vectors (q_s) between the parallel branches of d_{xy} and d_{yz} bands. The scattering wave vector q_s is thickness-dependent.

References

1. Tan S., *et al.* Interface-induced superconductivity and strain-dependent spin density waves in FeSe/$SrTiO_3$ thin films. *Nat. Mater.* **12**, 634-640 (2013).
2. Zhang Y., *et al.* Distinctive orbital anisotropy observed in the nematic state of a FeSe thin film. *Phys. Rev. B* **94**, 115153 (2016).
3. Li W., *et al.* Stripes developed at the strong limit of nematicity in FeSe film. *Nat. Phys.* **13**, 957-961 (2017).
4. Ren Z., *et al.* Nanoscale decoupling of electronic nematicity and structural anisotropy in FeSe thin films. *Nat. Commun.* **12**, 10 (2021).
5. Tan S.Y., *et al.* Observation of Dirac cone band dispersions in FeSe thin films by photoemission spectroscopy. *Phys. Rev. B* **93**, 104513 (2016).
6. Kivelson S.A., Fradkin E. & Emery V.J. Electronic liquid-crystal phases of a doped Mott insulator. *Nature* **393**, 550-553 (1998).
7. Blanco-Canosa S., *et al.* Resonant X-ray scattering study of charge-density wave correlations in $YBa_2Cu_3O_{6+x}$. *Phys. Rev. B* **90**, 054513 (2014).
8. Sato Y., *et al.* Thermodynamic evidence for a nematic phase transition at the onset of the pseudogap in $YBa_2Cu_3O_y$. *Nat. Phys.* **13**, 1074-1078 (2017).
9. Tang C., *et al.* Interface-enhanced electron-phonon coupling and high-temperature superconductivity in potassium-coated ultrathin FeSe films on $SrTiO_3$. *Phys. Rev. B* **93**, 020507 (2016).
10. Zhang W.H., *et al.* Effects of surface electron doping and substrate on the superconductivity of epitaxial FeSe films. *Nano Lett.* **16**, 1969-1973 (2016).
