# Supplementary material for: Incommensurate smectic phase in close proximity to the high-Tc superconductor FeSe/SrTiO3
Source: Nat Commun. 2021 Apr 13;12:2196. doi: 10.1038/s41467-021-22516-2 (PMC8044195; doi:10.1038/s41467-021-22516-2)
Supplement: Supplementary file 3 — Description of Additional Supplementary Files [file 41467_2021_22516_MOESM3_ESM.pdf]

Description of Additional Supplementary Files

File Name: Supplementary Movie 1
Description: Quasiparticle interference patterns of 3 UC FeSe at positive energy

File Name: Supplementary Movie 2
Description: Quasiparticle interference patterns of 3 UC FeSe at negative energy

File Name: Supplementary Movie 3
Description: Quasiparticle interference patterns of 2 UC FeSe at positive energy

File Name: Supplementary Movie 4
Description: Quasiparticle interference patterns of 2 UC FeSe at negative energy
