# Supplementary figures and images for: Incommensurate smectic phase in close proximity to the high-Tc superconductor FeSe/SrTiO3
Source: Nat Commun. 2021 Apr 13;12:2196. doi: 10.1038/s41467-021-22516-2 (PMC8044195; doi:10.1038/s41467-021-22516-2)

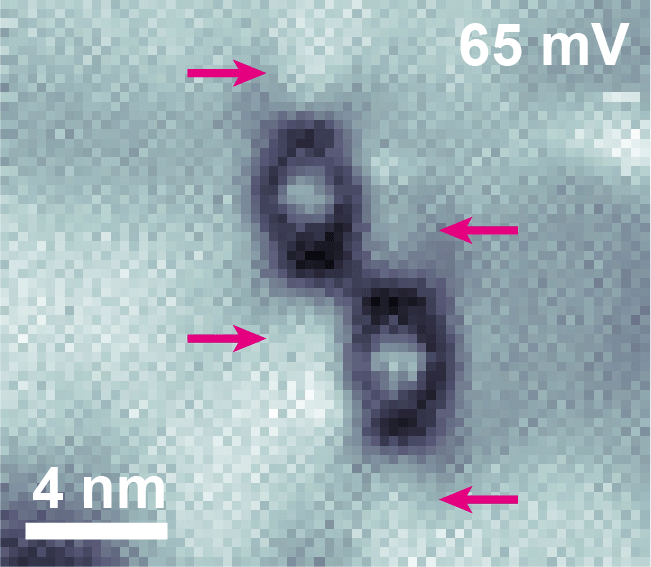

Supplement: Supplementary file 4 — Supplementary Movie 1 [file 41467_2021_22516_MOESM4_ESM.gif]

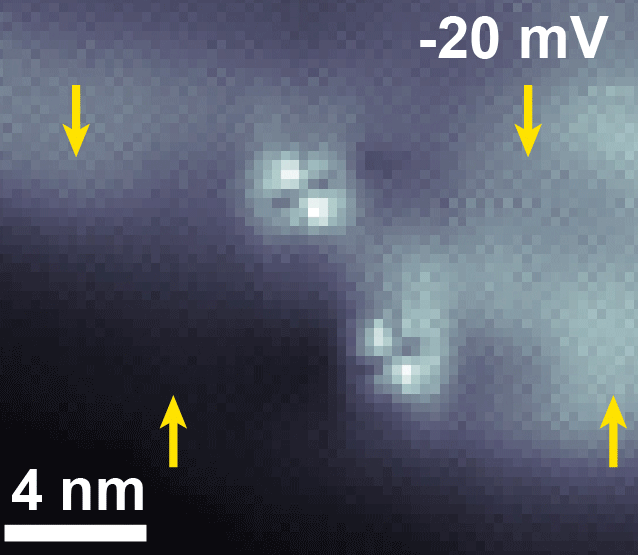

Supplement: Supplementary file 5 — Supplementary Movie 2 [file 41467_2021_22516_MOESM5_ESM.gif]

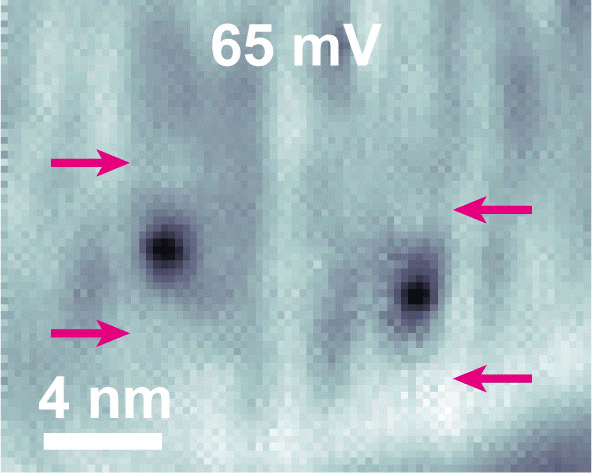

Supplement: Supplementary file 6 — Supplementary Movie 3 [file 41467_2021_22516_MOESM6_ESM.gif]

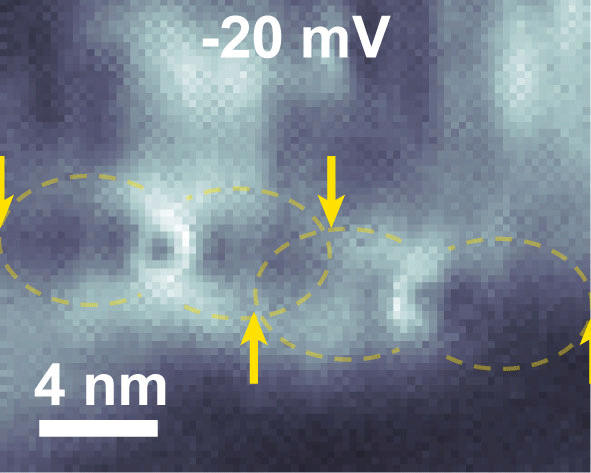

Supplement: Supplementary file 7 — Supplementary Movie 4 [file 41467_2021_22516_MOESM7_ESM.gif]
